# Case report: Successful treatment of ulcerative colitis-related post-colectomy enteritis refractory to multiple therapies with ustekinumab

**DOI:** 10.3389/fimmu.2024.1297508

**Published:** 2024-02-16

**Authors:** Chenglin Guo, Shengduo He, Huahong Wang

**Affiliations:** Department of Gastroenterology, Peking University First Hospital, Beijing, China

**Keywords:** ustekinumab, ulcerative colitis, post-colectomy, ulcerative colitis-related severe post-colectomy enteritis, high-output stoma

## Abstract

Ulcerative colitis-related severe post-colectomy enteritis is a rare condition. A few cases have undergone successful treatment with corticosteroids, Cyclosporine, Azathioprine, and Infliximab. We aim to evaluate the treatment outcome of ustekinumab in this rare case. Here we describe a 56-year-old woman with post-colectomy enteritis refractory to multiple therapies. Finally, the patient was administered with ustekinumab treatment. Under monitoring, the feces volume of the patient decreased from 5000-7000 mL per day to 1700-2000 mL. Over a one-year follow-up period, the patient gradually gained body weight, with the stoma drainage of formed brown stool. And the villi of the small intestinal mucosa restore growth. To our knowledge, this is the first report that indicates ustekinumab could be a treatment selection for ulcerative colitis-related severe post-colectomy enteritis.

## 1 Introduction

Colectomy is the optimal treatment strategy for patients with ulcerative colitis (UC) who develop intraepithelial neoplasia during disease surveillance. Although colectomy rates have decreased for the management of UC, surgery is still indicated for patients with severe UC, chronic refractory disease despite maximal/optimal medical treatment, or a dysfunctional colon (1). However, a few patients have enteritis postoperatively. UC-related enteritis is characterized by diffuse, superficial, ulcerative mucosal inflammation of the entire small bowel, with a typical onset after colectomy for UC (2, 3). The cause of post-colectomy enteritis remains unclear. It may result from the persistent inflammatory response, which could continue after colectomy, particularly in refractory UC cases. Other causes may include altered fecal stasis post-ileostomy, potential post-surgical ischemia and bacterial overgrowth (3). In 2004, Joel Rubenstein et al. first reviewed 11 cases of UC-related enteritis after colectomy and discussed the treatment options for this condition (4). Since then, several case reports have been published, all reporting an effective response to treatment by glucocorticoids, immunosuppressants or Tumor necrosis factor alpha (TNF-α) inhibitors (3, 5, 6). These patients were characteristic of massive intestinal bleeding, intestinal perforation and high-output stoma. In this report, we describe a patient with ulcerative colitis-related severe enteritis without consistent efficacy in recurrent stoma drainage under treatment with corticosteroids, infliximab and cyclosporine, which were sequentially utilized. In this case, ustekinumab, a monoclonal antibody against interleukin (IL)-12 and IL-23, finally curbed this intractable condition.

## 2 Case description

**Figure 1:**
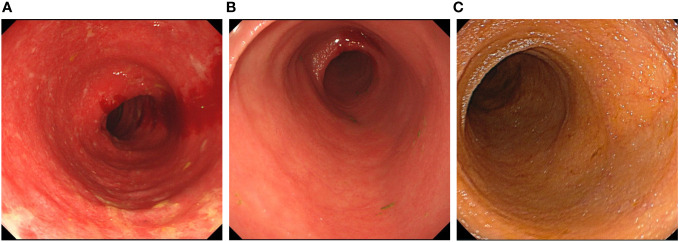
The endoscopy image in all stages of the treatment. **(A)** The colonoscopy before colectomy. The colonoscopy showed the mucosa was diffusely congested, with edema. Shallow ulcers were formed, and covered with white patches. The colonic bag disappeared, and the vein texture was unclear. **(B)** The ileoscopy after colectomy. The ileoscopy showed a disappearance of intestinal villi and an irregular vascular network. **(C)** The ileoscopy after treatment with ustekinumab.The ileoscopy showed intestinal villi was normal.

**Figure 2:**
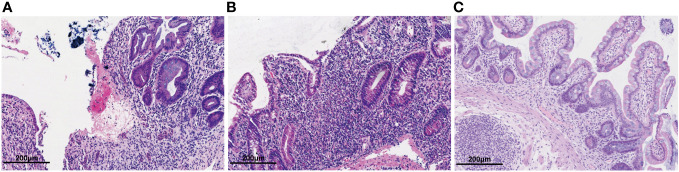
Hematoxylin-eosin staining of the intestinal mucosal. **(A)** Hematoxylin-eosin staining of the colonic mucosa before colectomy. Colon pathology showed mucosal erosion, ulcers, cryptitis and granulation tissue hyperplasia. **(B)** Hematoxylin-eosin staining of the intestinal mucosa after colectomy. Ileum pathology showed villus atrophy and shortening, mucosal erosion, cryptitis with irregular crypt structure and infiltrated lymphocytes and plasma cells. **(C)** Hematoxylin-eosin staining of the intestinal mucosa after after 1 year of ustekinumab treatment. Ileum pathology showed a reduction in lymphocytes and plasma cells compared to before, with regular crypt structures.

A 56-year-old woman diagnosed endoscopically and pathologically with UC was transferred to our hospital due to failed oral prednisone at 50 mg qd for 3 months for treating the relapsed condition of abdominal pain and bloody stools more than 10 times daily, which was effectively treated by methylprednisolone (40 mg ivgtt, qd) previously. Hence, steroid-resistant UC was diagnosed. She had been diagnosed with UC for 2 years before being admitted to our hospital. Her vital signs were stable, and physical examination showed epigastric tenderness without rebound pain. After excluding infections, vedolizumab at 300 mg was infused 3 times simultaneously with oral prednisolone at 40 mg qd and mesalazine 1g qid. However, bowel movements were not decreased. Under this circumstance, a colonoscopy was performed, revealing mucosal edema, congestion and erosion, accompanied by flake-like ulcers (**Figure 1A**). Histopathology showed chronic inflammation in the colonic mucosa, lamina propria edema, decreased number of glands, cryptitis and distortion of the crypt architecture (**Figure 2A**). No granuloma was found. In addition, an ileoscopy through the artificial stoma revealed a polypoid bulge in the sigmoid colon, and a biopsy suggested high-grade intraepithelial neoplasia. Considering the reduced effects of steroids and biologics, and the co-occurrence of a precancerous lesion, total colectomy + ileal pouch-anal anastomosis and a loop ileostomy were performed. In addition, ulcerative colitis and focal tubular adenoma grade I were confirmed by postoperative pathology.

**Figure 3:**
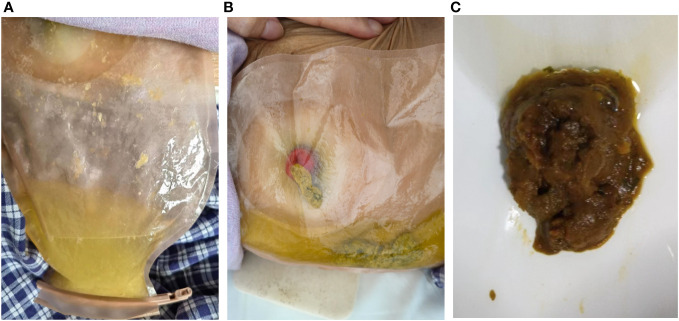
The stool traits before and after treatment with ustekinumab. **(A)** The yellowish watery excreta before treatment. **(B)** The formed stool after ustekinumab treatment. **(C)** The stool from stoma was still formed over a one-year follow-up.

However, three months postoperatively the patient was readmitted with intermittent abdominal pain and the characteristic of high-output ileostomy with frequent loose stools. On admission, daily stoma drainage was monitored at 5000-7000 mL/d of yellowish watery excretion (**Figure 3A**), with 2000 mL/d intake orally. Laboratory tests were negative for *Clostridium difficile*, fungi, cytomegalovirus or Epstein-Barr Virus. Endoscopy showed disappeared small intestinal villi and an irregular vascular network (**Figure 1B**). Pathological findings at 3 cm, 15 cm and 45 cm before the stoma confirmed local active inflammation of the ileal mucosa with numerous lymphocytes, plasma cells and some eosinophils infiltrated in the lamina propria. Focal cryptitis of distorted shapes and reparative hyperplastic glands were observed (**Figure 2B**). No epithelioid granuloma was found, neither vasculitis nor infection. Therefore, post-colectomy enteritis was suspected.

Consequently, hydrocortisone at 200 mg qd by intravenous infusion was administered for 7 days in combination with growth hormone at 0.4 mg qd by subcutaneous injection to promote the growth of small intestinal villi, and stoma drainage was reduced to <1000 mL/d for a time. However, during the decrement period, the stoma drainage increased again to 4000-6000 mL per day (2000 mL/d intake orally). Therefore, hydrocortisone at 200 mg qd was intravenously infused again but with no effect. Re-examination by endoscopy through the stoma for 20 cm showed atrophic intestinal villi, with the disappeared vascular network. Biopsy pathology was consistent with the condition of ineffective treatment.

Given that enteritis was refractory to steroids, and the serum levels of TNF-α were elevated to 10.10 pg/mL. 300 mg of Infliximab (IFX) was intravenously infused 2 times, and stoma drainage was reduced to 1000-2000 mL. Unfortunately, 1 month later, the drainage volume increased again to 5000-8000 mL/d, thus a third infusion of IFX at 300 mg was provided. Although the serum drug trough concentration was >20 µg/mL and anti-drug antibody levels were <4 ng/mL under the therapeutic drug monitoring, the loss of response occurred again and the drainage did not decrease. Cyclosporine A at 50 mg qd by intravenous infusion was then administered, and the dose was gradually increased to 150 mg qd according to the blood concentration. Nevertheless, bowel movements did not decrease significantly. Worse, liver damage occurred, so cyclosporine A was discontinued.

Ustekinumab has been used for the treatment of ulcerative colitis and may be effective for post-colectomy enteritis. Eventually, ustekinumab at 260 mg (body weight 33.5 kg) was used for the treatment of this enteritis refractory to multiple therapies. Twenty days after the drug was administered, the drainage volume started to decrease.

Despite adequate enteral nutrition support, the nutritional status remained poor, body mass index was 13.6 kg/m2 and albumin was 27 g/L. To avoid the low concentration of ustekinumab induced by hypoalbuminemia and cachexia, ustekinumab was administered at 4-week intervals. Under monitoring, the feces volume from the stoma was about 1700-2000 mL/d, with an oral intake of 2200-2600 mL/d.

**Figure 4:**
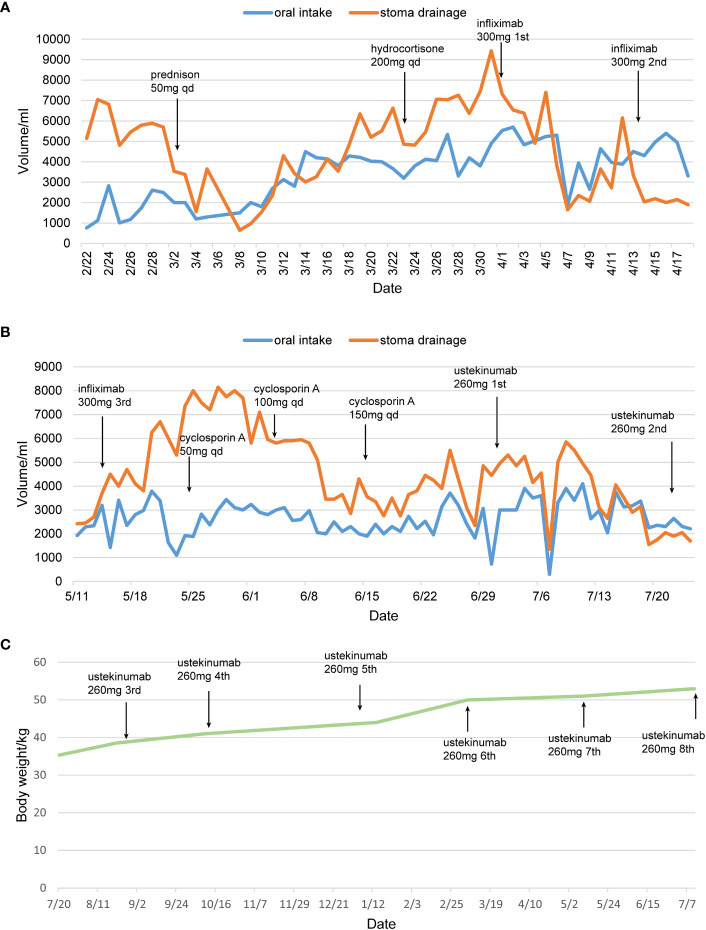
The therapeutic timeline of the patient. **(A, B)** Treatment protocol and changes in drainage volume. **(C)** Changes of body weight after treatment with ustekinumab. qd, *quaque die*. The patient was discharged from April 19^th^ 2022 to July 7^th^ 2023.

Two months later, the patient received additional ustekinumab without any adverse event. The drainage volume from the stoma is 1000-1800 mL/d with oral intake of 2000- 2600 mL/d, and the stool trait was mushy and formed (**Figure 3B**). Thereafter, the patient was treated with ustekinumab 260mg every 8 weeks. Over a one-year follow-up period, the patient gradually gained body weight, with the stoma drainage of formed brown stool (**Figure 3C**). And repeat enteroscopy showed that the villi of the small intestine were generally normal (**Figure 1C**). Hematoxylin-eosin staining of the intestinal mucosa revealed a decrease in lymphocytes and plasma cells compared to before (**Figure 2C**). The main treatment steps for the patient are shown in **Figures 4A–C**.

## 3 Discussion

For severe medically refractory ulcerative colitis, colectomy such as total colectomy + ileal pouch-anal anastomosis is the ultimate treatment solution. However, after colectomy, around 0.8% of patients might develop very severe and sometimes fatal enteritis (6). In 2021, Kohyama et al. defined this type of enteritis as ulcerative colitis-related severe enteritis (UCRSE) (6). Briefly, individuals with massive intestinal bleeding, intestinal perforation, high-output stoma, or a need for potent medications, including prednisolone, immunosuppressants, immunomodulators, and/or biologics, were included. The rare case described here could be defined as a UCRSE, and represents the first reported UCRSE successfully treated by ustekinumab to our knowledge.

The treatment of UCRSE remains difficult with limited evidence. Intravenous corticosteroids are recommended as the initial standard treatment option for acute severe UC in several guidelines, as this treatment induces clinical remission and reduces mortality (7–10). For UCRSE, corticosteroids were also most frequently used as a first-line therapy to relieve symptoms rapidly (11). Corporaal et al. reviewed 42 cases with UC-related enteritis, virtually all of whom were either cured or controlled, especially with high intravenous doses of corticosteroids (5). In this case, the patient responded well with 200 mg qd intravenous doses of corticosteroids at first, but relapsed after decrement. The patient then developed into a steroid refractory case though the dose of corticosteroids was increased but with no effect.

IFX was effectively used in several steroid-refractory UCRSE cases. In a patient with mild bleeding from UC-related enteritis, 5-aminosalicylic acid and corticosteroids did not confer complete recovery, who eventually responded well to IFX treatment with no progressive worsening (12). In another case, IFX was ineffective for colitis but improved postoperative enteritis and led to endoscopic healing (11). Considering this evidence, IFX was administered to the patient after corticosteroids. Since the patient had extensive small intestinal and colonic lesions, IFX might leak into the stool and not reach sufficient blood levels, we adopted an accelerated IFX treatment strategy with 300 mg twice in one month (13). After infusion, stoma drainage was reduced from 4000-6000 mL to 1000-2000 mL daily with IFX tough concentration reaching 20 pg/mL. Unfortunately, when administered the third infusion, the loss of response was developed. Cyclosporine was then administered to improve the condition but failed, so we decided to switch to a biologic with another mechanism of action.

Ustekinumab, a monoclonal antibody targeting the p40 subunit of IL-12 and IL-23, has been approved for the treatment of UC in several countries. The UNIFI trial demonstrated the superiority of ustekinumab over placebo in inducing and maintaining remission in patients with UC, not only in naïve patients but also in those with bio-failure (14). Some real-world studies have verified the effect of ustekinumab in UC (15–17). The pathogenesis of UCRSE remains unclear, of which surgical stress and genetic susceptibility may be vital factors. In a previous study, intestinal flora and epithelial gene expression changed toward a colon-like pattern in the remnant small intestine after colectomy (18). A secondary change of the T-helper (Th) cell ratio in peripheral CD4+ T cells may be associated with the pathophysiology of small intestinal inflammation (19). Since IL-12/IL-23p40 antagonist could suppress Th1, Th17 and Th22 activation to alleviate intestinal inflammation, we decided to administer ustekinumab after failed IFX treatment (20). Diarrhea remission was achieved rapidly after one intravenous dose. The case was accompanied by cachexia during treatment, so we escalated the dose of the biological agent. Intensification strategies for ustekinumab include shortening the interval between the treatments to 4 or 6 weeks and/or intravenous reinduction (18). Considering cachexia could lead to large amounts of drug loss, a second intravenous dose was administered 4 weeks later to maintain the effective concentration of ustekinumab (21). This resulted in stool formation and gradually achieved oral intake recovery. Subcutaneous ustekinumab was administered at 8-week intervals to maintain long-term remission after two intravenous doses.

The strength of this case report is that it is the first to describe the potential usefulness of ustekinumab for UCRSE. It provides us with a new treatment selection for those UCRSE patients who are refractory to other therapies. Despite the encouraging results herein described, we should be aware that this is an isolated clinical case, only one patient was studied, and a cause-effect relationship cannot be confirmed. Besides, the IL12/23 change after administration of ustekinumab was not evaluated at the intestine or circulation.

In conclusion, UCRSE is a rare but severe and potentially fatal complication of colectomy, requiring early diagnosis and treatment. The case presented above shows the first reported success of ustekinumab in accelerating treatment in corticosteroid- and IFX-refractory UCRSE, providing a new treatment option for such patients. Meanwhile, more studies are warranted to evaluate dosing escalation strategies, and more patients with UCRSE treated with ustekinumab are required to evaluate long-term effectiveness and safety.

## 4 Patient perspective

The patient and her families were satisfied with the treatment and expressed their willingness to follow up for a long time. At first, the patient was apprehensive about the new treatment plan, worrying that ustekinumab might not be effective. However, as the treatment progressed, the patient began to experience noticeable improvements. The drainage volume from the stoma decreased, bowel movements gradually became more formed, and body weight started to increase. This had a positive impact on patient’s daily life, enabling her to engage in routine self-care. Additionally, throughout the therapy with ustekinumab, the patient did not encounter any significant side effects, which left the patient highly satisfied.

## Data availability statement

The raw data supporting the conclusions of this article will be made available by the authors, without undue reservation.

## Ethics statement

The studies involving humans were approved by Ethics Committee at Peking University First Hospital. The studies were conducted in accordance with the local legislation and institutional requirements. The participants provided their written informed consent to participate in this study. Written informed consent was obtained from the individual(s) for the publication of any potentially identifiable images or data included in this article.

## Author contributions

CG: Writing – original draft. SH: Writing – original draft. HW: Supervision, Writing – review & editing.
